# Impact of B-Scan Averaging on Spectralis Optical Coherence Tomography Image Quality before and after Cataract Surgery

**DOI:** 10.1155/2017/8148047

**Published:** 2017-05-23

**Authors:** Dominika Podkowinski, Ehsan Sharian Varnousfaderani, Christian Simader, Hrvoje Bogunovic, Ana-Maria Philip, Bianca S. Gerendas, Ursula Schmidt-Erfurth, Sebastian M. Waldstein

**Affiliations:** Christian Doppler Laboratory for Ophthalmic Image Analysis, Vienna Reading Center, Department of Ophthalmology, Medical University of Vienna, Spitalgasse 23, 1090 Vienna, Austria

## Abstract

**Background and Objective:**

To determine optimal image averaging settings for Spectralis optical coherence tomography (OCT) in patients with and without cataract.

**Study Design/Material and Methods:**

In a prospective study, the eyes were imaged before and after cataract surgery using seven different image averaging settings. Image quality was quantitatively evaluated using signal-to-noise ratio, distinction between retinal layer image intensity distributions, and retinal layer segmentation performance. Measures were compared pre- and postoperatively across different degrees of averaging.

**Results:**

13 eyes of 13 patients were included and 1092 layer boundaries analyzed. Preoperatively, increasing image averaging led to a logarithmic growth in all image quality measures up to 96 frames. Postoperatively, increasing averaging beyond 16 images resulted in a plateau without further benefits to image quality. Averaging 16 frames postoperatively provided comparable image quality to 96 frames preoperatively.

**Conclusion:**

In patients with clear media, averaging 16 images provided optimal signal quality. A further increase in averaging was only beneficial in the eyes with senile cataract. However, prolonged acquisition time and possible loss of details have to be taken into account.

## 1. Introduction

Spectral-domain optical coherence tomography (SD-OCT) is the most important imaging modality for diagnosis and management of retinal diseases [1]. Recent technical advances have significantly improved the image resolution, contrast, scanning speed and angle, depth penetration, and automated analysis techniques in OCT imaging [2]. In order to allow precise identification of fine clinical details and robust automated segmentation, improvements in image quality are a main focus of research in OCT technology.

One important reason for low image quality in OCT is speckle noise, that is, random granular noise [3]. Based on the assumption that noise must be randomly distributed in an image, averaging of several images of the same object can be applied to enhance image quality [4]. Some current OCT devices include an image averaging function in their software and are able to provide averaged images based on the scanning protocol selection. If combined with the built-in eye tracking technology, images of the same retinal location can be acquired several times and image averaging of identical retinal regions is feasible. This concept, termed “automated real time averaging” (ART), is widely used in clinical practice and research studies. However, there are no solid data or guidelines available regarding the impact of averaging extent on image quality. Inadequately selected averaging settings may result in unnecessarily poor image quality, patient overexposure, or inefficient use of resources.

To allow a comprehensive evaluation of image quality characteristics, standardized parameters measuring the various aspects of image quality are required. Frequently used parameters for quantitative analysis are the signal-to-noise ratio (SNR) and, as a surrogate variable, the reproducibility of retinal thickness measurements. Moreover, some studies rely on subjective grading for the assessment of image quality [5,6]. However, using subjective scores can be variable even for trained experts.

Most retinal diseases occur with exceeding frequency in elderly patients, a patient group where media opacities, in particular cataract, are common [7]. Previous studies have demonstrated that cataract can affect the OCT image quality as well as the reproducibility of retinal thickness measurements [5,8-10]. However, these studies did not consider the role of image averaging.

The purpose of our study was to establish optimal image averaging settings in retinal OCT acquisition based on objective parameters for the assessment of image quality. Furthermore, we evaluated the optimal settings for image averaging in patients with and without cataract.

## 2. Patients/Materials and Methods

This study was a prospective, observational, noninterventional case series. The study protocol was prospectively approved by the Ethics Committee of the Medical University of Vienna. All study procedures complied with the tenets set forth in the Declaration of Helsinki. Prior to inclusion, all patients provided written informed consent to participate.

Patients with the diagnosis of senile cataract and planned cataract surgery at the Medical University of Vienna, Austria, were included into the study between March and October 2013. Exclusion criteria were retinal pathologies, which could influence central fixation, and optical media abnormalities apart from cataract that could influence imaging quality. Only one eye per patient was included.

### 2.1. Examination and Imaging Procedure

A complete ophthalmic examination, including slit lamp examination and fundus biomicroscopy, was performed in all patients one week before and one week after uncomplicated cataract surgery. All surgeries were performed by a single, experienced surgeon (CS) using clear corneal or scleral corneal incisions, phacoemulsification, and implantation of an acrylic intraocular lens into the capsular bag. Preoperatively, cataracts were classified according to the Lens Opacities Classification System III (LOCS III) as nuclear (N), cortical (C), or posterior (P) [11]. If the LOCS III score was >N3, <C3, and <P3, the cataract was categorized as nuclear; if C was equal to or higher than 3, it was categorized as cortical; and if P was equal to or higher than 3, the categorization was posterior [5].

**Figure 1:**
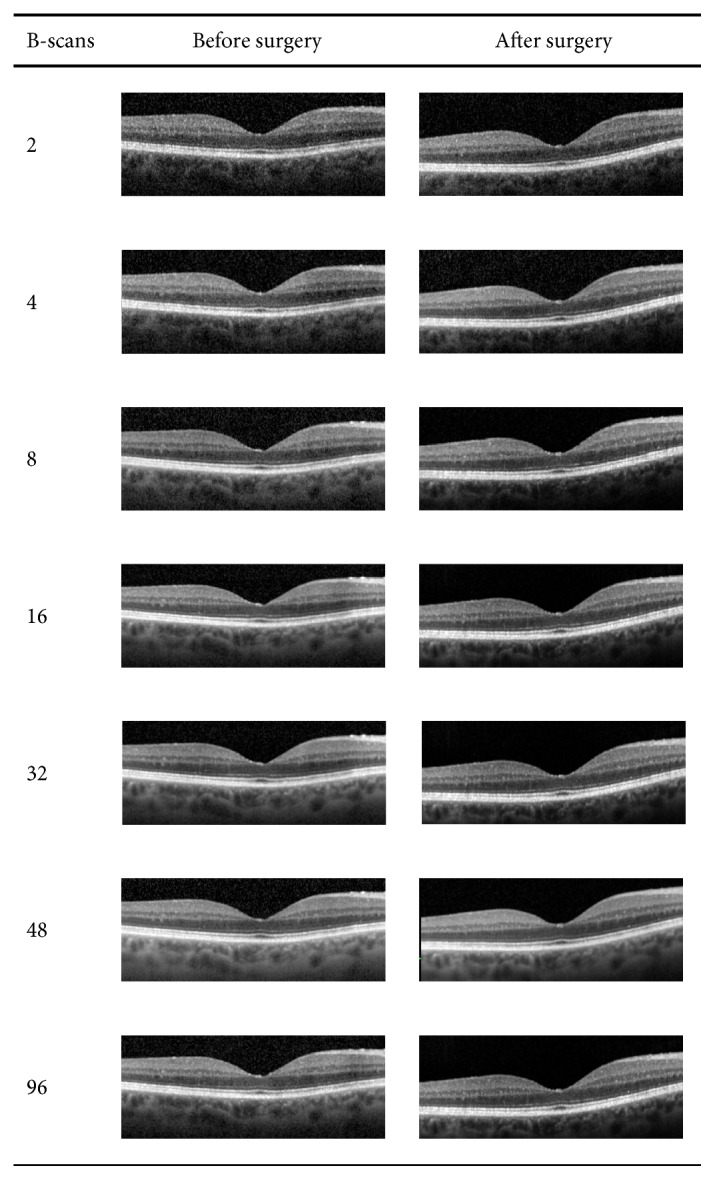
Exemplar OCT scans of a study patient acquired with different averaging settings. Left: number of B-scans averaged. Middle: images acquired before cataract surgery with turbid media. Right: images acquired after cataract extraction.

After pupil dilatation, OCT scans were acquired at each visit following a standardized acquisition protocol by a single investigator (DP). The Spectralis™ HRA+OCT (Heidelberg Engineering, Dossenheim, Germany) was used to scan repeated patterns of 512 A-scans in a single horizontal line of 6 mm length corresponding to an angle of 20°. For each repetition, the automated real-time averaging (ART) feature was employed with systematically varying the number of averaged B-scans as follows: The degree of averaging was selected according to a logarithmically escalating scale with seven steps using 2, 4, 8, 16, 32, 48, and 96 frames. Thus, seven scans were acquired at each visit for each patient. The order of scans was randomized to counteract potential systematic bias. The first scan acquired was set as a reference, and the built-in device follow-up function was used to align all following scans (before and after surgery) to the identical retinal position. All scans were exported as TIFF files for further processing as shown in Figure 1.

**Figure 2:**
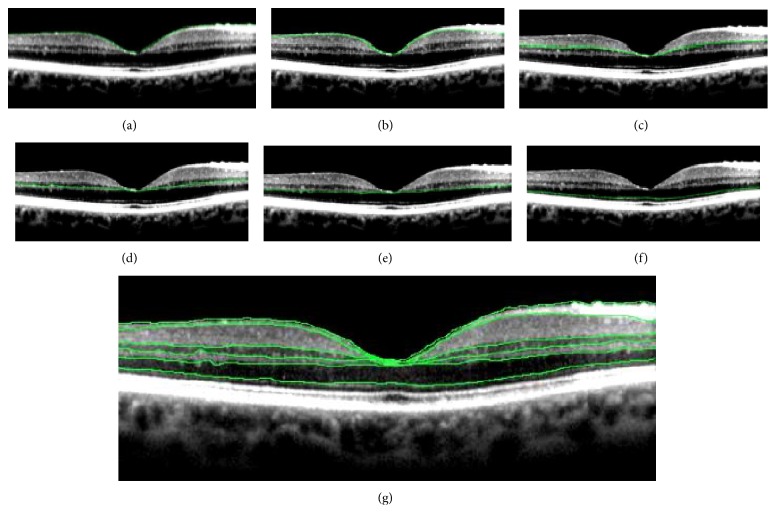
Example of manually annotated borders of the individual retinal layers on an optical coherence tomography scan. (a) Border between vitreous and NFL, (b) border between NFL and GCL, (c) border between IPL and INL, (d) border between INL and OPL, (e) border between OPL and ONL, (f) border between ONL and ELM, and (g) all annotated borders. Annotations were performed on each scan with different averaging settings; thus, on 7 scans preoperatively and 7 scans postoperatively for each patient.

To be able to assess the contrast and distinction between the individual retinal layers, the following layer boundaries were manually annotated by a single investigator (DP) in Adobe Photoshop version CS6 (Figure 2): boundaries between vitreous and nerve fiber layer (NFL), border between nerve fiber layer and ganglion cell layer (GCL), border between inner plexiform layer and inner nuclear layer (INL), border between inner nuclear layer and outer plexiform layer (OPL), border between outer plexiform layer and outer nuclear layer (ONL), and border between outer nuclear layer and external limiting membrane (ELM). Reproducibility data on the annotation procedure has been reported previously [12].

### 2.2. Image Quality Quantification

Three different measures were employed for quantitative image quality analysis: [1] signal-to-noise ratio (SNR), [2] Cohen's *d* value, and [3] automated layer segmentation performance. All computations were performed with the MATLAB software (version R2013a, The MathWorks Inc., Natick, Massachusetts, USA). The measures are defined as follows.

#### 2.2.1. Signal-to-Noise Ratio (SNR)

The SNR compares the level of a desired signal in the image to the level of noise. It is computed individually for each manually annotated intraretinal layer as

(1) $$\begin{array}{c} SNR=\frac{\mu_{l}}{\sigma_{l}}, \end{array}$$

where *μ*_*l*_ and *σ*_*l*_ are the mean and the standard deviation of the grey values within a single layer. Under the assumption of signal and noise being uncorrelated, the SNR is expected to increase with the square root of the number of averaging frames, resulting in a favorable appearance of the image. The SNR of an image was computed by averaging the SNR values of all the layers.

#### 2.2.2. Cohen's *d* Value

The dissimilarity of individual retinal layers based on their grey values was assessed by measuring the overlap of the image intensity distributions between neighboring manually annotated layers. It is measured using Cohen's *d* value, which is inversely proportional to the overlap of distributions, and it is computed for two neighboring layers *L*_1_ and *L*_2_ as

(2) $$\begin{array}{c} d\left(L_{1},L_{2}\right)=\frac{\mu_{l1}-\mu_{l2}}{\sqrt{\left(\left(N_{l1}-1\right)\sigma_{l1}^{2}+\left(N_{l2}-1\right)\sigma_{l2}^{2}\right)/\left(N_{l1}+N_{l2}-2\right)}}, \end{array}$$

where *μ*_*l*1_, *σ*_*l*1_^2^, and *N*_*l*1_ are the mean, the variance, and the sample size of an intensity distribution that are generated from pixels forming layer *L*_1_(resp., for *L*_2_). A high value of Cohen's *d* indicates that the neighboring layer intensity distributions are well separated and thus their distinction is high. For each manually annotated layer, the *d* value was computed as the average of the two *d* values with its two neighboring layers above and below. The Cohen's *d* value of an image was computed by averaging the values of all the layers.

#### 2.2.3. Automated Layer Segmentation Performance

The ability to differentiate between the individual retinal layers based on their appearance was further assessed by measuring the performance of an automated segmentation method in correctly segmenting each layer. The automated segmentation method is based on pixel classification. Segmentation of a layer was computed by first learning Gaussian mixture model representation of its intensity distribution as well as those of the two neighboring layers. Each intensity distribution was modeled using three Gaussian components. Then, the pixels were classified to a particular layer by maximum likelihood criteria. Accuracy of automated segmentation method is evaluated with the Dice index, which computes the similarity between two sets of pixels corresponding to automatically segmented layer *S*_auto_ and the manually denoted one *S*_manual_ (taken as the ground truth) as

(3) $$\begin{array}{c} Dice\left(S_{auto},S_{manual}\right)=2\frac{\left|S_{auto}\cap S_{manual}\right|}{\left|S_{auto}\right|+\left|S_{manual}\right|}. \end{array}$$

The Dice index is in the range [0,1] with higher values denoting better segmentations. It was computed separately for each layer. The Dice index of an image was computed as the average of all the layers.

### 2.3. Statistical Analysis

Statistical analysis was performed using the SPSS V.22 statistical package (IBM CROP, Armonk, NY, USA). The impact of the varying degrees of averaging on the three different image quality measures was assessed by descriptive statistics and performed separately for preoperative and postoperative acquisitions shown in the histograms and graphs using different matrices described in the results section. In addition, we computed for each eye which degree of preoperative averaging reached the quality of the postoperative setting.

## 3. Results

Thirteen eyes of 13 patients were included. Nine participants were female and five were male. The mean ± standard deviation patient age was 68.3 ± 8.7 years. Eight of the cataracts were classified as nuclear, four as cortical, and one as posterior. The classification is based on the highest score in one of the three categories according to the LOCS III grading. All included cataracts were of mixed type. For each eye, 5 individual layers with 6 boundaries were annotated on each baseline and follow-up scan using 7 different averaging settings. This resulted in a total of 14 B-scans, 70 individual layers, and 84 layer boundaries that were analyzed for each patient. In total, 1092 layer annotations were performed.

### 3.1. Signal-to-Noise Ratio

Preoperatively, a seemingly linear increase in SNR was seen with escalating averaging settings, with an increase in SNR at each averaging increment.

**Figure 3:**
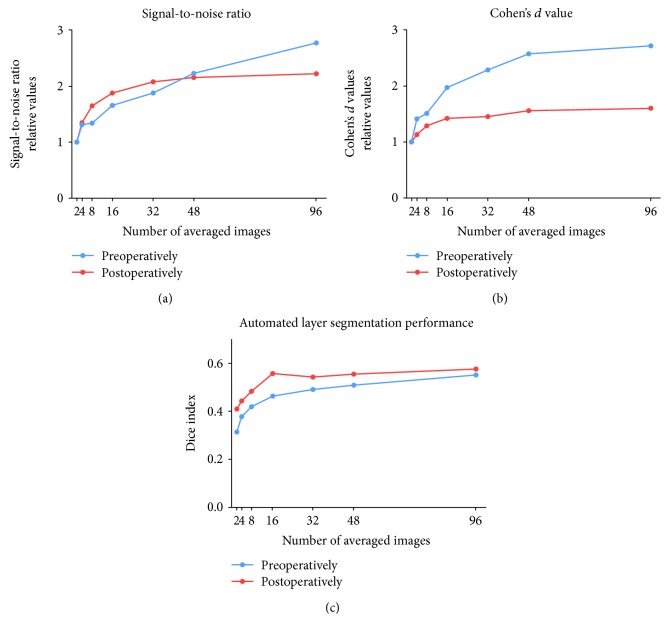
Quantitative measures of image quality. The graphs show signal-to-noise ratio (SNR; (a)), the Cohen's *d* value (b), and intraretinal layer segmentation performance (c). SNR and Cohen's *d* are computed relative to the baseline at two averaged frames. The SNR compares the level of a desired signal in the image to the level of noise. Cohen's *d* value describes the dissimilarity of individual retinal layers based on their grey values and is inversely proportional to the overlap of distributions. It is computed for two neighboring layers. The ability to differentiate between the individual retinal layers based on their appearance was further assessed by measuring the performance of an automated computer algorithm on correctly segmenting each layer based on pixel classification. Cohen's *d* value and the automated layer segmentation performance are demonstrated as relative values. Results are shown as mean for each averaging frame increment, separately for pre- and postoperative results overall patients and all layers.

**Figure 4:**
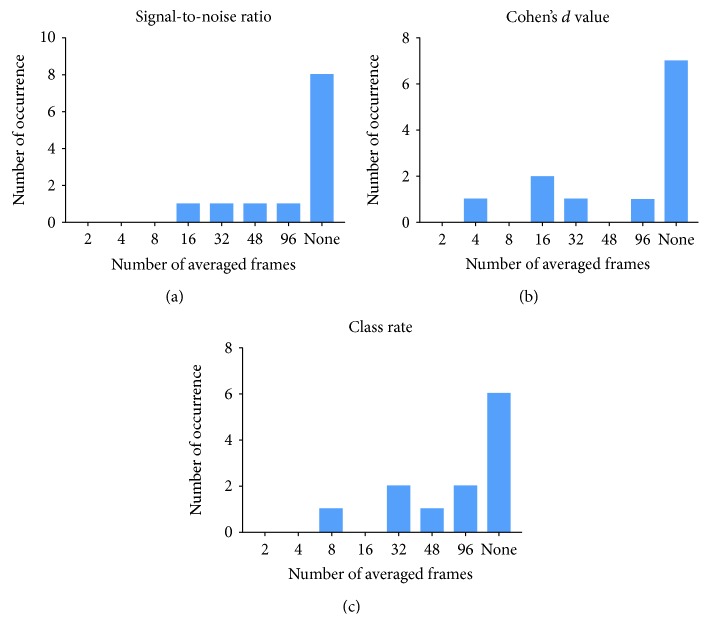
Intraindividual evaluation of quantitative measures of image quality for all 13 patients. On the *x*-axis, the number of averaged frames preoperatively is shown, which is needed to achieve the same result as an average of 16 frames postoperatively. A 16-frame scan provides the most beneficial setting after cataract surgery. Comparison is computed individually for each patient. On the *y*-axis, the number of cases is shown. “None” indicates that there was no averaging setting preoperatively, which achieved the same result as postoperatively.

Postoperatively, an increase was detected until 16 averaged frames. Thereafter, a further increase in averaging did not result in SNR changes, resembling a logarithmic growth pattern with saturation at 16 averaged frames (Figure 3). Preoperatively, all the eyes required equal or more averaged frames to achieve the same levels of SNR as 16 averaged frames postoperatively (Figure 4).

### 3.2. Cohen's *d* Value

The differentiation of the individual retinal layers based on the overlap of image intensity distribution as measured by Cohen's *d* value increased logarithmically pre- and postoperatively, with progressive saturation achieved at 48 averaged frames (Figure 3). Preoperatively, all but one eye required more averaged frames to achieve the same Cohen's *d* value as 16 averaged frames postoperatively, shown in Figure 4.

### 3.3. Layer Segmentation Performance

The ability to segment the individual retinal layers as measured by the Dice index showed a strongly logarithmic growth pattern both preoperatively and postoperatively (Figure 3). Preoperatively, the Dice index saturated at 48 averaged frames. Postoperatively, the increase in the Dice index was substantial from two to 16 averaged frames, with only minimal improvement with further increasing image averaging. Preoperatively, all but one eye required more averaged frames to achieve the same segmentation performance as 16 averaged frames postoperatively (Figure 4).

## 4. Discussion

This study provides evidence for the selection of optimal image averaging settings in retinal OCT acquisition based on objective parameters for the assessment of image quality in patients with and without cataract. In the eyes with clear optical media, image averaging using 16 frames resulted in high levels of SNR and good distinction between the individual retinal layers. A further increase in the degree of image averaging did not provide additional benefit in image quality. On the other hand, in the eyes with senile cataract, higher degrees of image averaging allowed a further increase in image quality and may therefore be clinically useful. Our findings may be of relevance for the selection of OCT scanning protocols in clinical research and practice. If the image quality achieved with higher averaging settings is similar to the image quality at lower averaging settings, it is beneficial to use the settings which are quicker to acquire based on time and compliance; therefore, a lower number of averaging is to favor.

Few studies have addressed the impact of B-scan averaging on image quality in retinal OCT previously. A retrospective study by Pappuru et al. demonstrated significantly improved distinction between the retinal layers using image averaging in healthy eyes [13]. While scan averaging at four frames resulted in a significant improvement of image quality, an increase to 20 frames showed no significant change. Similar results were reported in a study by Sakamoto et al. [14]. These previous findings are corroborated by our study for the eyes with clear optical media; however, in the eyes with cataract, our study demonstrated that further averaging may be of value for image quality. B-scan averaging had no significant influence on RNFL thickness measurements in one study, although another study demonstrated superior reproducibility of RNFL measurements by averaging 100 frames [15,16]. In both studies, only patients with clear media were included.

Several studies investigated the influence of senile cataract on the image quality of retinal OCT. van Velthofen et al. used a subjective grading of the scans prior to surgery as well as the objective parameter signal strength and SNR to determine the image quality changes before and after cataract surgery [5]. The objective image quality parameters improved postoperatively, similarly to our study. The largest influence on the image quality was demonstrated for nuclear cataract; although due to the small study size, the statistical subgroup analysis was inconclusive. An analysis of the different subtypes of cataracts was not within the scope of our study, also due to limitations in the sample size.

The other available studies focused mainly on the reproducibility of retinal thickness measurements as a surrogate variable for image quality relating to cataract surgery. A study by Bambo et al. demonstrated improved reproducibility of thickness measurements with clear optical media. Preoperatively, reproducibility was superior using Spectralis OCT compared to Cirrus OCT, presumably due to the eye tracking function in Spectralis OCT [17]. Similar results were shown by other published studies, where patients with different diseases, such as diabetes, retinitis pigmentosa, and multiple sclerosis, were included [18-20].

In our study, we employed three different measures for the assessment of image quality in retinal OCT: the SNR and two different measures to assess the ability to distinguish between the individual retinal layers, that is, Cohen's *d* value and layer segmentation Dice index. All three measures are well-established in image analysis. In clinical practice, particularly the ability to discern fine anatomical detail is critically important for the evaluation of imaging biomarkers for disease management [21]. In clear optical media, optimal levels of these two parameters were achieved by averaging 16 image frames. Furthermore, high image averaging settings result in prolonged acquisition times, which may lead to unnecessarily lengthy visits, reduced patient compliance, and compromised image quality. Higher degrees of image averaging may lead to excessive smoothing of the image. Such smoothing of the Spectralis OCT image may be caused by the repeated scanning not capturing the identical anatomical position and may result in unwanted loss of morphologic detail [22].

Based on the data provided by our study, a setting of 16 averaged frames seems to provide a solid balance between the advantages and disadvantages of image averaging in patients with clear optical media. Concerning eyes with optical media opacities with an increase in image averaging may result in a good balance regarding the investigated image quality parameters.

Even more for clinical practice, an evidence-based selection of OCT scanner settings is particularly relevant in large scale multicenter clinical trials. The advent of automated large-scale three-dimensional computerized analysis of OCT data highlights the critical need for standardized scanning protocols. In the future, population-level research may become increasingly relevant with the establishment of large publicly available image data bases consisting of highly standardized data repositories.

This study is mainly limited by its sample size. However, it should be noted that an extensive scanning protocol was used and laborious manual annotations of several individual retinal layers were undertaken. This resulted in 1092 annotations and data points for analysis, allowing solid conclusions. Furthermore, our results show a slight decrease in Cohen's *d* value at 32 averaged frames postoperatively. This unexpected decrease may be associated with ocular surface changes as a consequence of cataract surgery. This diagnosis is to be expected after surgery [23]. The order of scan acquisition with the different image averaging settings was randomized in our study to avoid confounding effects of dry eye disease. A further potential limitation of this study is the lack of annotations for the photoreceptor and retinal pigment epithelium (RPE) layers. However, we expect OCT signal properties to be stable across the retinal structures at least in healthy cases.

In conclusion, our study suggests that 16 averaged frames may provide optimal image quality in the examination of patients with clear optical media by Spectralis OCT. Excessive image averaging did not deliver corresponding image quality benefits, led to disadvantageous smoothing effects, and should be avoided in clinical practice. However, in patients with media opacities, such as senile cataract, a higher degree of image averaging may result in a further increase in image quality. Our results may be of value in designing standardized OCT scanning protocols in clinical practice and future clinical trials.
